# Effect of endoscope flexibility on tissue dissection profile assessed with pulsed water jet device: ensuring safety, efficacy, and handling of thin devices for neuroendoscopic surgery

**DOI:** 10.1186/s13104-021-05475-1

**Published:** 2021-02-17

**Authors:** Tetsuya Kusunoki, Tomohiro Kawaguchi, Atsuhiro Nakagawa, Yuta Noguchi, Shin-Ichiro Osawa, Hidenori Endo, Toshiki Endo, Ryuta Saito, Masayuki Kanamori, Kuniyasu Niizuma, Teiji Tominaga

**Affiliations:** Department of Neurosurgery, Tohoku University Graduate School of Medicine, 1 Seiryo-machi, Aoba-ku, Sendai, Miyagi 980-8574 Japan

**Keywords:** Minimally invasive surgery, Endoscopic surgery, Incurvation, Pulsed water jet

## Abstract

**Objective:**

We developed an actuator-driven pulsed water jet device (ADPJ) for flexible neuroendoscopy to achieve effective tissue dissection with vasculature preservation. Although flexibility is a strong advantage for minimally invasiveness, the effect of the ductile curvature on the dissection profiles remains unknown. The purpose of this study was to clarify the impact of the curvature change of the ADPJ connecting tube on the dissection safety and efficacy.

**Results:**

Three ADPJ connecting tubes with different inner diameters (1.0, 0.75, 0.5 mm) were used to dissect the brain phantom. They were bent at 3 angles: 0°, 60°, and 120°. The dissection profiles were evaluated using the mean depth and coefficient of variation (CV) for efficacy and safety, respectively.The larger inner diameter connecting tube dissected more deeply. The dissection depth was not changed regardless of the curvature degree in each tube. There was no significant difference in CVs regardless of inner diameter and curvature. The ductile curvature of the flexible neuroendoscope did not affect the efficacy and safety of the ADPJ dissection profile. Among the numerous instruments, tube-formed devices, including suction and injecting devices such as ADPJ, can be used safely and effectively without flexibility-related limitations.

## Introduction

Minimal invasiveness is the most important social requirement for medical examination and surgery. Flexible endoscopy is considered minimally-invasive for procedures across all medical fields, including gastrointestinal or colorectal examinations [1–3]. Target lesions can be approached and dissected with specially designed instruments, such as wired forceps or tubes, which are inserted via the working channel, by rotating, grasping, or pulling them. Although flexibility is the biggest advantage of the endoscope for approaching the deep-located lesions, some clinicians have reported that the movement of the devices can be hindered when the endoscope is bent significantly.

**Fig. 1:**
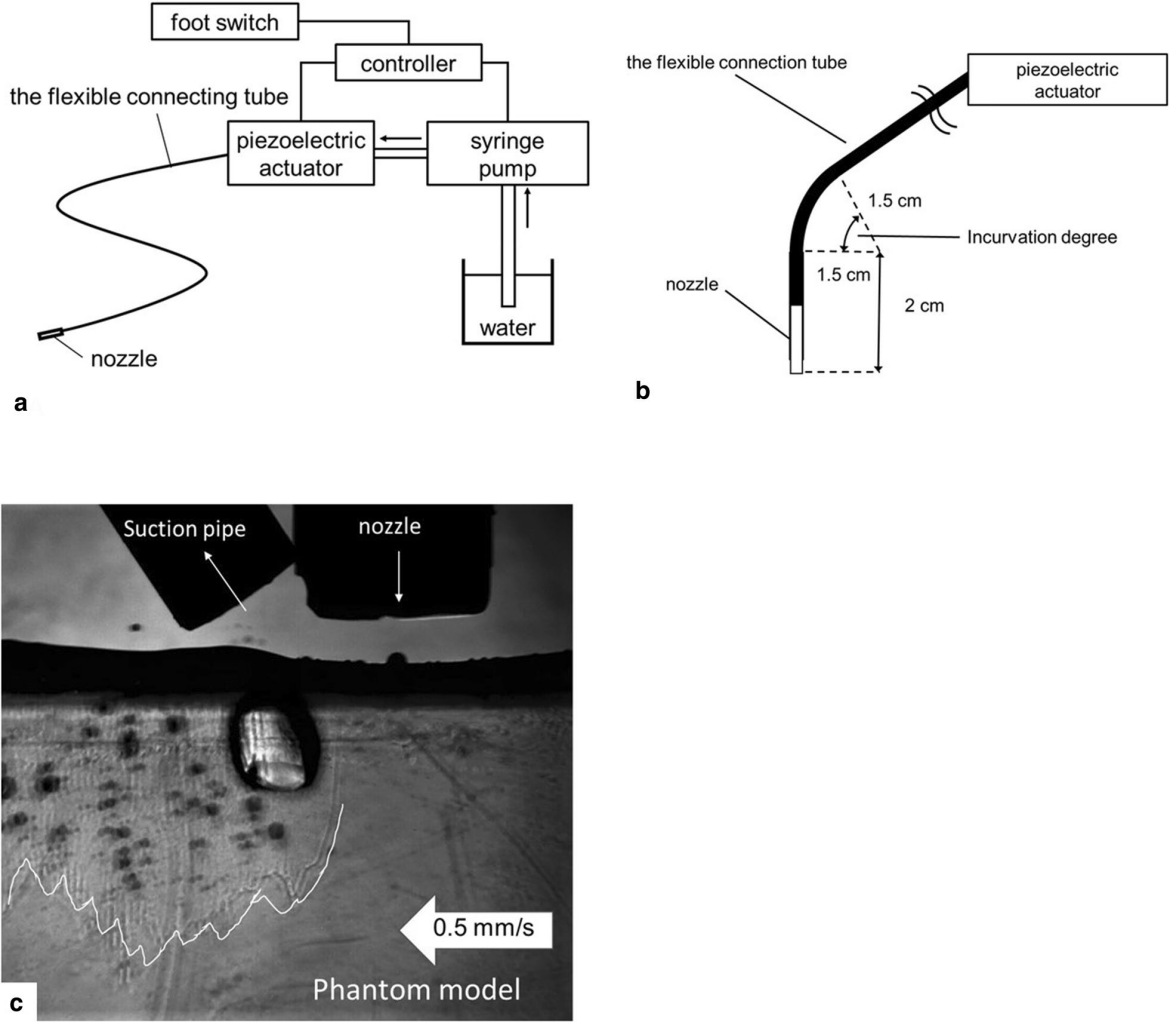
**a** Scheme of the ADPJ and the flexible connecting tube. **b** Scheme of the flexible connecting tube. **c** Photograph showing brain phantom dissection with ADPJ

Flexible neuroendoscopes became available for neurosurgery recently, resulting in a signfinicant reduction of brain retraction and damage to surrounding normal structures when approaching deep-located lesions. On the other hand, owing to the small approach corridor, the number of tools that can be used is restricted. Therefore, it is difficult to achieve effective hemostasis during sharp dissection with flexible neuroendoscopy. Our group has developed an actuator-driven pulsed water jet device (ADPJ) [4]. Ejected water jet can dissect soft tissues, but vessels cannot be penetrated because of the elasticity. This tissue selectivity based on the tissue-specific physical properties is the significant characteristics of water jet dissection. The classical water jet devices which has been used for liver and kidney surgeries since 1980s [5–9], and there were several issuses to be soled due to large amount of water usage, such as water splash and tissue debris dispersion. Unlike them, ADPJ utilizes pulsed water resulting in increased dissection rate, reduced blood loss, and functional preservation durig glioma and pituitary surgeries [10–12]. We have developed the ADPJ device for flexible neuroendoscopy to facilitate a further reduction in surgical invasiveness (Fig. 1). A preclinical feasibility study revealed ADPJ device for flexible neuroendoscopy can dissect the ventricle wall with vessel preservation [13]. During neurosurgery with a flexible neuroendoscope, the connecting tube of the ADPJ bends during endoscope motion. Although flexibility is a strong advantage, the effect of the ductile curvature of a flexible neuroendoscope on the efficacy and safety of the ADPJ dissection profiles remains unknown.

This study clarifies the impact of changes in the curvature of the ADPJ connecting tube. We investigated the dissection profile of ADPJ with different degrees of curvature of the connecting tube to confirm its efficacy and safety. We discuss how the flexibility of the endoscope affects the handling of surgical tools such as elongated suction and injecting tubes inserted via the small channel of the endoscope, which has not been evaluated before.

## Main text

### Method

#### The actuator-driven pulsed water jet device (ADPJ) for flexible neuroendoscopy

In the present study, a flexible neuroendoscope (CLV-260, VEF TYPE V; Olympus) was used. The flexible connecting tube, which was made of polyetheretherketone resin, was attached to the piezoelectric actuator unit to generate a pulsed water jet (Fig. 1a). The nozzle, with width of 1.6 mm and a 0.15-mm hole, was placed at the distal end of the connecting tube. The entire length and the outer diameter of the connecting tube were 65 cm and 1.6 mm, respectively. In this study, 3 types of connecting tubes with different inner diameters (1.0, 0.75, 0.5 mm) were used.

#### Water flow rate optimization

For each connecting tube, the flow rate of the water supply had to be optimized. The optimal flow rate was defined as the rate at which the stable pulsed water jet ejection could occur without an overflow. A high-speed camera (NX4S2, IDT Japan Co., Ltd.) was set in front of the nozzle and operated at a frame rate of 25,000 frames/s to observe the ejected pulsed water jet. For this experiment, the water flow rate ranged from 2.0 to 3.0 ml/min. The other settings of the actuator in the present study were as follows: drive input voltage of 30 V and frequency of 200 Hz.

#### Brain phantom profiles

Brain phantoms were created with gelatin for in vitro experiments. Gelatin (Nitta Gelatin; BCN300S) 3.5 wt % (gelatin weight/total weight) was dissolved in warm water and cooled to solidify at 280 K in an acryl cell. Their breaking strengths were measured with a compact tabletop universal tester (EZ-TEST EZ-S, Shimadzu Co., Ltd.) before experimental use to confirm that their elasticities were similar to that of the brain parenchyma [13].

#### Incurvation the connecting tube

To assess the impact of its degree of curvature on the dissection profile, the connecting tube was bent in 3 different angles: 0°, 60°, and 120°. The radius of curvature was 1.5 cm, and the pivot point was set at 2 cm away from the nozzle; the other segment of the connecting tube was kept straight (Fig. 1b).

#### Dissecting the brain phantom

The brain phantom was placed on a moving table (EZSM 3E020K, Oriental Motor Co., Ltd.), and the nozzle was set 1.5 mm perpendicularly away from the target. The pulsed water jet was ejected from the nozzle while the table was moved horizontally at 0.5 mm/s. The suction tip was placed next to the nozzle of the ADPJ to control water splash and to evacuate pooled water within the dissecting area (Fig. 1c). The suction pipe size and the condition setting were not varied through this study. The dissection depth was recorded with a high-speed camera and measured at 21 points at a 1-mm interval. The dissection profiles were evaluated using the mean depth and coefficient of variation (CV), for efficacy and safety, respectively [14]. The CV was calculated as the standard deviation divided by the mean of the dissection depths.

#### Statistic analysis

To obtain statistical data, all experiments were repeated 5 times. All quantitative data are presented as mean ± standard deviation. The Tukey–Kramer post hoc test was performed using the JMP Pro 15 software (SAS Institute Inc.). Statistical significance was set at *p* < 0.05.

### Result

#### Optimal water supply flow rate of ADPJ

**Fig. 2:**
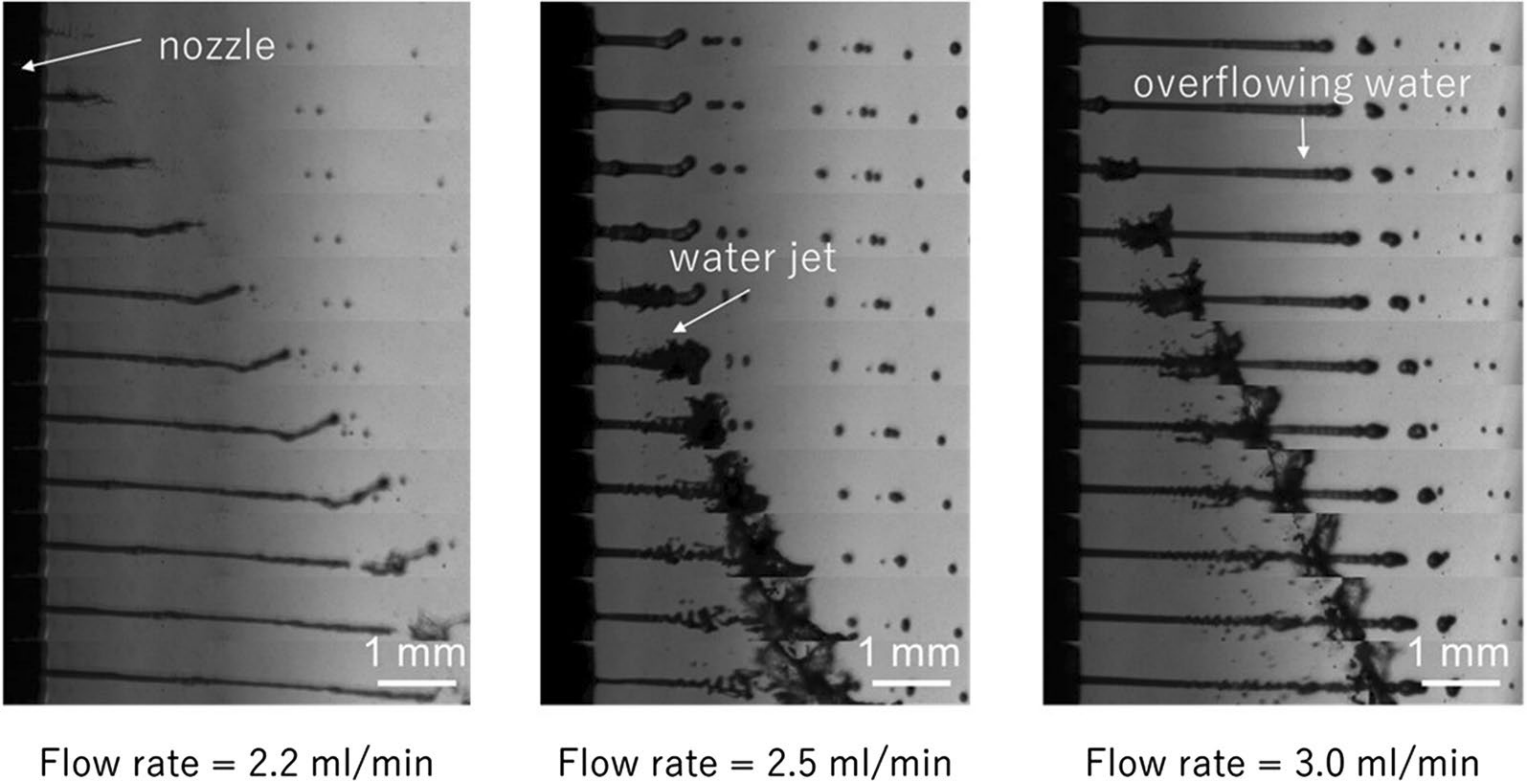
Sequential photographs of the ejected pulsed water jet with different water flow rates. The left panel shows insufficient water jet generation. The middle panel shows sufficient water jet generation. The right panel shows overflowing water blocking the generated water jet. No suction pipe was used for this experiment. The nozzle with 1.6 mm width was used for all experiments

To obtain the optimal setting, the pulsed water jet was ejected at different flow rates and the outcomes were recorded. A detailed analysis revealed that the ideal pulsed water jet formation was observed with a different flow rate for each connecting tube (Fig. 2). No suction pipe was used for this experiment. The optimal water flow rate of the connecting tube was 2.9 ml/min for an inner diameter of 1.0 mm, 2.5 ml/min for 0.75 mm, and 2.3 ml/min for 0.5 mm. These settings were used in the present study.

#### Dissection profile and the inner diameter of the connecting tube

**Fig. 3:**
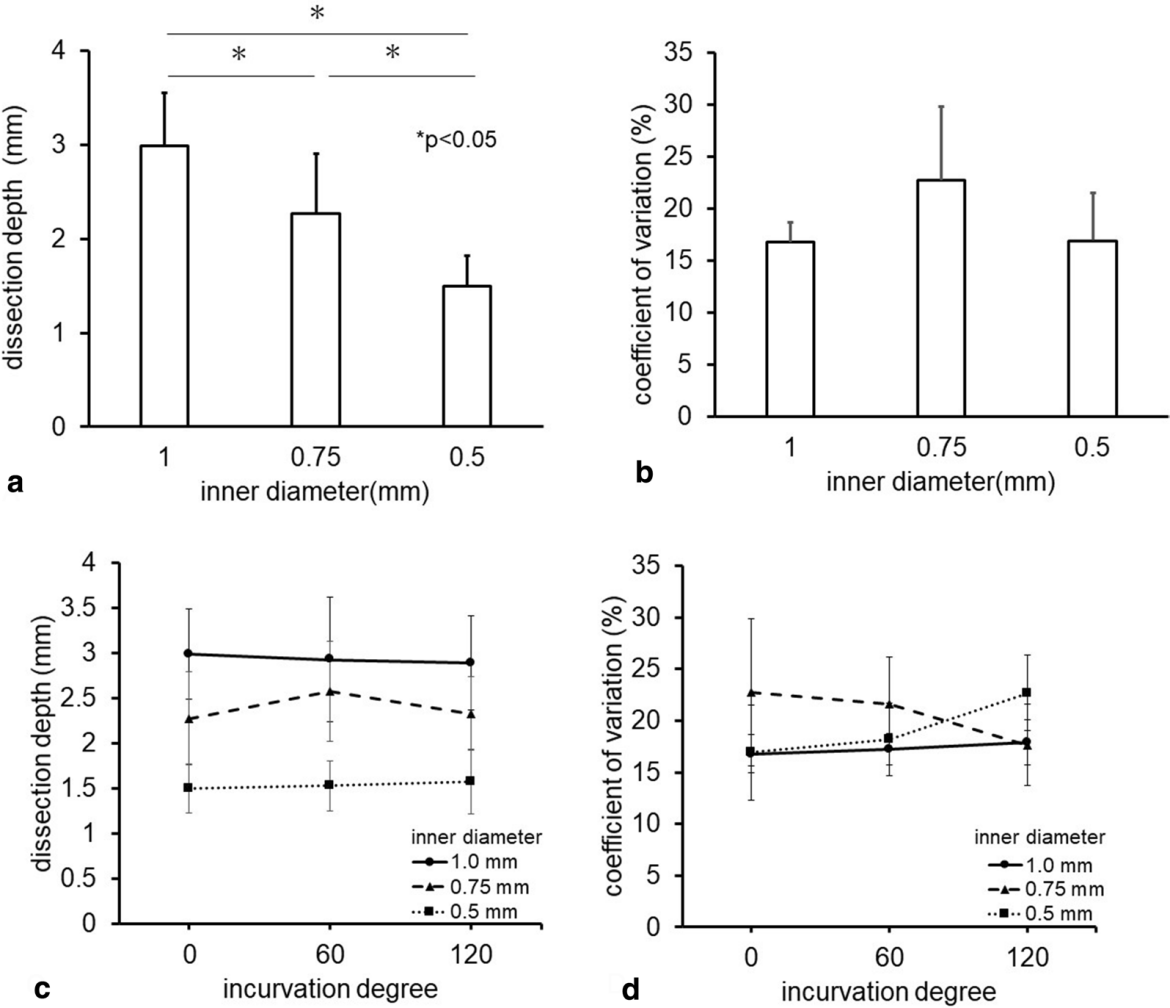
**a** Graph showing the comparison of the dissection depths for the 3 types of connecting tubes with different inner diameters. Asterisks show statistical significance (*p* < 0.05). **b** Graph showing the comparison of the coefficients of variation (CVs) of the dissection depths of the 3 connecting tubes with different inner diameters. There is no statistically significant difference. **c** Graph showing the comparison of the dissection depths for the different degrees of curvature of the connecting tubes. There is no statistically significant difference. **d** Graph showing the comparison of the coefficients of variation (CVs) of the dissection depths for the different degrees of curvature of each connecting tube. There was no statistically significant difference

To investigate the effect of the inner diameter of the connecting tube on the dissection profile, the brain phantom was dissected by the pulsed water jet generated with 3 different connecting tubes. The dissection was deepest (2.99 ± 0.56 mm) with the largest-diameter tube (1.0 mm) and shallowest (1.50 ± 0.32 mm) with the smallest-diameter tube (0.5 mm) (Fig. 3a). Although the CV slightly varied from 16.8% to 22.8% with the different inner diameters, there was no statistically significant difference (Fig. 3b).

#### Dissection profile and curvature of the connecting tube

To investigate the effect of the degree of curvature of the connecting tube on the dissection profile, the brain phantom was dissected by the pulsed water jet generated using the 3 different connecting tubes. Three different angles (0°, 60°, and 120°) were assessed for each tube. The dissection depth of the 0.75-mm diameter connecting tube increased by 13.2% with a 60-degree curvature, but it was not statistically significant (Fig. 3c). Similarly, a slight change in dissection depth was observed with 2 other connecting tubes, but there was no significant difference, regardless of the degree of curvature. Although the CVs of the dissection depths ranged from 16.8% to 22.8% for each tube with the different degrees of curvature, there was no statistically significant difference (Fig. 3d).

### Discussion

At present, the number of commercially available flexible neuroendoscopes is limited, and their maximum working angles are 90° both anteriorly and posteriorly. Our results showed that ADPJ can be used effectively under clinical conditions.

In the present study, the optimal flow rate was peculiar to each connecting tube. Because the inner diameters of the 3 connecting tubes were different, the amount of water inside the tube was distinct. Insufficient water supply resulted in “blank shooting,” and excessive water supply unexpectedly resulted in excess water, which blocked the subsequently generated water jet. As shown in Fig. 3a, the pulsed water jet ejected via the connecting tube with a large inner diameter was found to dissect more deeply. This may be attributable to the amount of water; a larger amount of water could penetrate the target more deeply. This result indicates that the inner diameter of the connecting tube can affect the ADPJ dissection profile.

On the other hand, the ductile curvature of the connecting tube did not affect the dissection profile of the ADPJ. Even if the curvature is intricate, the loss of energy may be minimal. This is similar to peripheral arterial blood pressure waveform monitoring through the intra-arterial catheter. A small indwelling needle is introduced into a peripheral artery and connected to a semi-soft tube filled with normal saline. The pulsating waveform and blood pressure are measured at the distal end of the tube because the tube is thick and sufficiently hard for transmitting the pressure. Likewise, our ADPJ connecting tube was sufficiently hard, and the deformation by curvature or pressure exertion may have been minimized to conduct the pulsed water jet.

The CV is a statistical measure of the dispersion of data points in series data around the mean, representing the ratio of the standard deviation to the mean. Because the CV shows the extent of variability, it characterizes the dispersion of dissection depths in our study. Regarding the inner diameter of the connecting tube, the dissection depth was deeper with a larger inner diameter. The CVs ranged from 16.8% to 22.8% for the connecting tubes with different inner diameters, and there was no statistical significance. Regarding the tube curvature, CVs ranged from 16.8% to 22.8% for each tube, and there was no statistically significant difference. Taken together, the tissue dissection ability of ADPJ is stable and safe regardless of the inner diameter or degree of curvature of the connecting tube.

## Conclusion

In conclusion, the ductile curvature of the flexible neuroendoscope did not affect the efficacy and safety of the ADPJ dissection profile under clinical conditions. Among the numerous instruments, tube-formed devices, including suction and injecting devices such as ADPJ, can be used safely and effectively without flexibility-related limitations.

## Limitations

Our study has several limitations. First, only three variations of inner diameter were studied due to material limitations. Because the channel of the neuroendoscope was small, the variation in the tube size was not very large. Second, this study was conducted in air. Surgical maneuvers with neuroendoscopes may be performed in cerebrospinal fluid, and the optimal setting for ADPJ and the behavior of the ejected pulsed water jet may differ. To overcome these issues, further investigation is required.

## Data Availability

The datasets used and/or analyzed during the current study are available from the corresponding author on reasonable request.

## Abbreviations

ADPJ: Actuator-driven pulsed water jet device
CUSA: Cavitron ultrasonic surgical aspirator
CV: Coefficient of variation
